# Well-concealed advanced duodenal carcinoma with Muir–Torre syndrome: a case report and review of literature

**DOI:** 10.1186/s40792-023-01603-0

**Published:** 2023-02-13

**Authors:** Tomoyuki Sugi, Osamu Shimomura, Shinji Hashimoto, Kazuhiro Takahashi, Manami Doi, Yoshihiro Miyazaki, Tsuyoshi Enomoto, Yoshimasa Akashi, Kazuhisa Araki, Tatsuya Oda

**Affiliations:** Department of Gastrointestinal and Hepato-Biliary-Pancreatic Surgery, Faculty of Medicine, University of Tsukuba, 2-1-1 Tennodai, Tsukuba, Ibaraki 305-8576 Japan

**Keywords:** Muir–Torre syndrome, Duodenum carcinoma, Partial duodenectomy, Pancreas-sparing duodenectomy, Genetic screening testing

## Abstract

**Background:**

Muir–Torre syndrome is an autosomal-dominant mutation in mismatch repair genes that gives rise to sebaceous tumors and visceral malignancies over time. Because colorectal and genitourinary cancers are common in Muir–Torre syndrome, duodenal carcinoma diagnoses are often delayed.

**Case presentation:**

A 58-year-old woman presented with severe emaciation, anorexia, and upper abdominal pain. She had a history of rectal carcinoma, ascending colon carcinoma, and a right shoulder sebaceous carcinoma. Upper gastrointestinal endoscopy and computed tomography examinations suggested duodenal obstruction due to superior mesenteric artery syndrome, leading to long-term observation. Seven months later, she was finally diagnosed with duodenal carcinoma of the third portion. As the papilla of Vater was preservable due to tumor location, she received a partial duodenectomy in lieu of a pancreatoduodenectomy. Pathologically, the tumor was a well-differentiated adenocarcinoma with a classification of T3N0M0 Stage IIA (UICC, 8th edition). The postoperative course was uneventful and her appetite returned. A mutation in mismatch repair gene MSH2 confirmed the diagnosis of Muir–Torre syndrome genetically. Three years later, her nutritional status has fully recovered and she is free from both recurrence and metastasis.

**Conclusion:**

In patients with comorbid skin sebaceous tumors and gastrointestinal malignancies, genetic screening is strongly recommended. Patients with Muir–Torre syndrome require long-term follow-up, and function-preserving treatment is desirable.

## Background

Autosomal-dominant Muir–Torre syndrome (MTS) is a rare condition caused by germline mutations in the DNA mismatch repair mechanism and is a phenotypic variant of Lynch syndrome. MTS is characterized by simultaneous appearance of both visceral malignancies and sebaceous tumors over time [1], with colorectal and genitourinary malignancies as the most common finding in 51% and 24% of cases, respectively [2]. Here, we describe a case of advanced duodenal carcinoma manifesting with severe malnutrition, in an MTS patient with a well-concealed malignancy.

## Case presentation

A 58-year-old, emaciated woman with a body mass index (BMI) of 17.1 kg/m^2^ (153.0 cm height/40.0 kg weight) presented with anorexia, nausea, and upper abdominal pain. In addition to a family history of colorectal cancer, the patient had a prior history of multiple gastrointestinal tumors, including rectal carcinoma (20 years old) and ascending colon carcinoma (39 years old), plus a right shoulder sebaceous carcinoma (48 years old). While a diagnosis of MTS was possible based on a history of a cutaneous tumor and visceral malignancies, she had yet to be diagnosed.

**Fig. 1:**
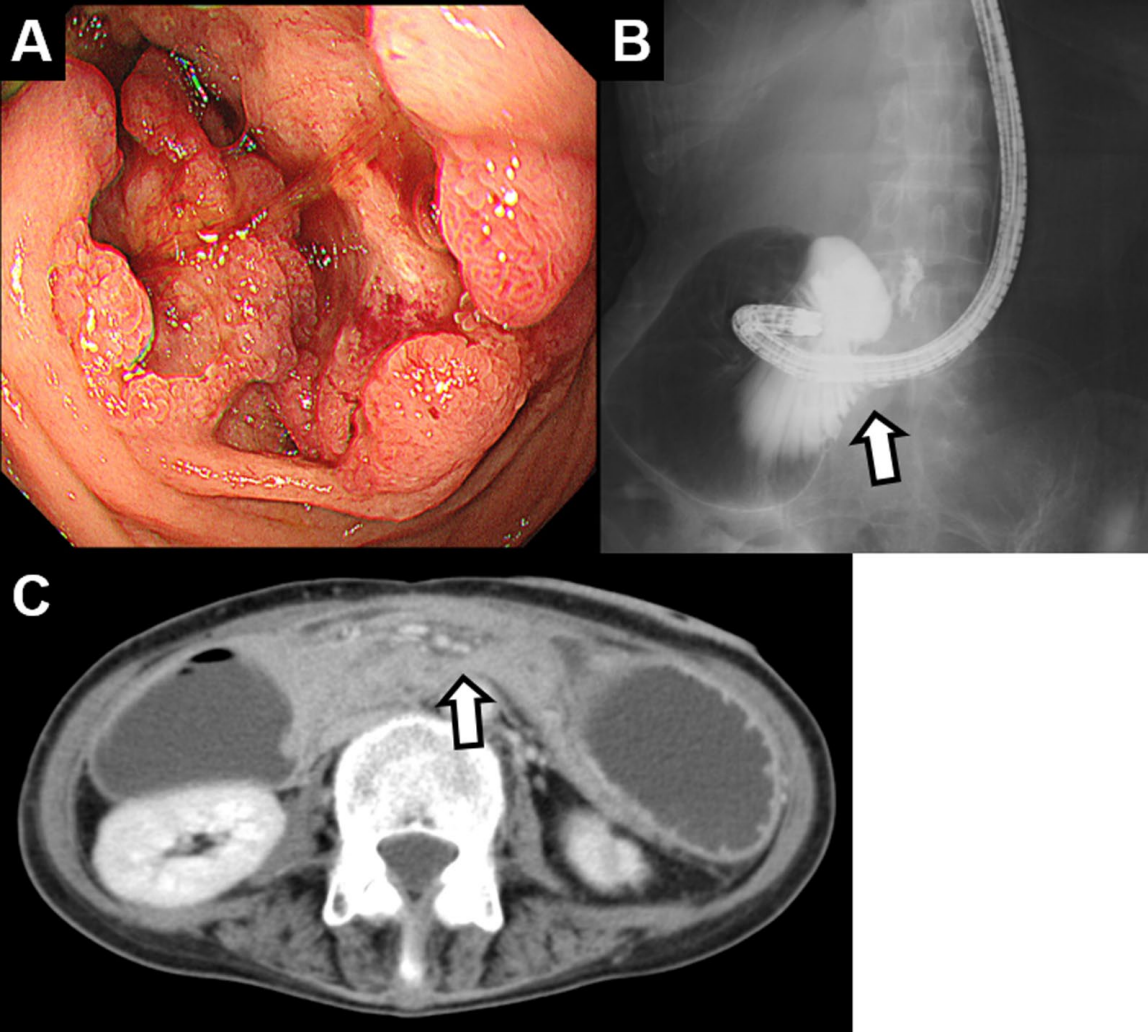
**A** Upper gastrointestinal endoscopy revealing a type 2 tumor in the third duodenal portion. **B** Upper gastrointestinal series revealing that contrast medium was unable to pass the third duodenal portion. **C** Computed tomography revealing the stricture in the third duodenal portion, but no mass could be identified. The stomach and duodenum are shown dilated

Even though tumors were not discovered during upper gastrointestinal endoscopy and computed tomography examinations suggested duodenal obstruction due to superior mesenteric artery syndrome, she was placed under long-term observation. Malnutrition developed 7 months after symptoms first appeared, with BMI decreasing to 12.0 kg/m^2^ (28.0 kg body weight). An upper gastrointestinal endoscopy redo revealed an ulcerative tumor in the third portion of the duodenum that pathology diagnosed as an adenocarcinoma (Fig. 1A). An upper gastrointestinal series then revealed that contrast medium was unable to pass the third portion of the duodenum (Fig. 1B) and computed tomography revealed a stricture in that area (Fig. 1C). The stomach and duodenum were dilatated (Fig. 1C). She was diagnosed with clinical T3N0M0 Stage IIA duodenal carcinoma (UICC, 8th edition).

**Fig. 2:**
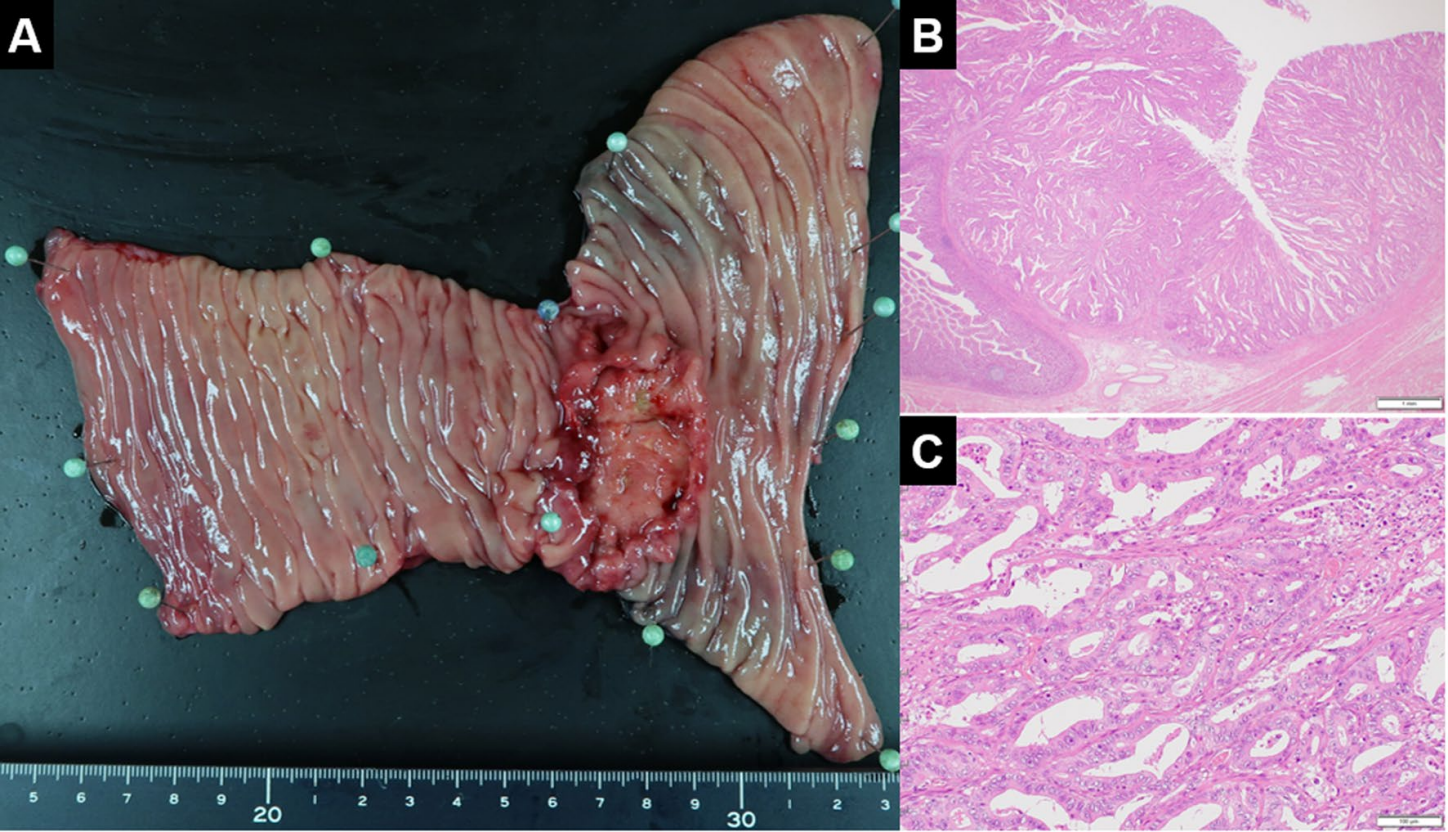
**A** The tumor was 60 × 35 mm in size in the third duodenal portion. **B**, **C** Pathological tests revealed that the carcinoma originated from the duodenal membrane and was well differentiated

**Fig. 3:**
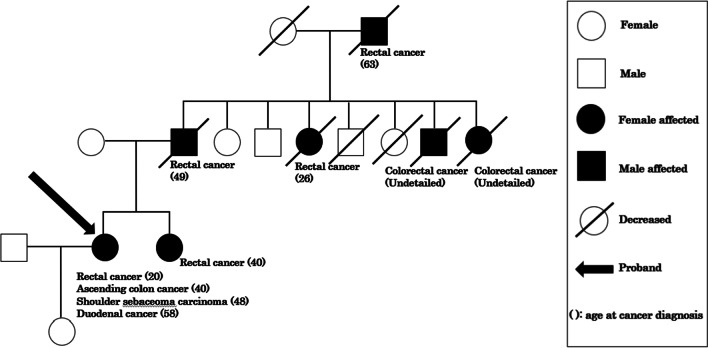
The pedigree chart of the patient

Considering her general condition, a partial duodenectomy was performed because the tumor location was amenable to preservation of the papilla of Vater. Only the para-duodenal lymph nodes were dissected. The operation lasted 305 min with a blood loss volume of 100 ml. The tumor was 60 × 35 mm in size and histopathological examination confirmed a well-differentiated adenocarcinoma (T3N0M0 Stage IIA [UICC, 8th edition]) (Fig. 2A–C). Microsatellite instability of the tumor was high. Genetic testing revealed defects in the DNA mismatch repair (MMR) gene MSH2 and confirmed the diagnosis of Muir–Torre syndrome. The pedigree tree showed that the patient had one first-degree relative, one second-degree relative, and three third-degree relatives with colorectal cancer (Fig. 3).

The postoperative course was uneventful and good appetite was recovered with no upper abdominal symptoms after surgery. However, nutritional recovery was poor in the early postoperative period, probably due to prolonged abstinence from food. Considering her general condition and regimen of adjuvant chemotherapy for gastric cancer [3], tegafur/gimeracil/oteracil was selected as postoperative adjuvant chemotherapy. After 3 months, adjuvant chemotherapy was discontinued at her request. Three years later, her nutrition status remains fully recovered and she is free from both recurrence and metastasis.

## Discussion

MTS was first independently described by Muir et al. in 1967 and Torre in 1968 [4, 5]. The genes implicated in MTS (MSH2, MSH6, MLH1, MYH1, and PMS2) predispose individuals to sebaceous tumors and visceral malignancies that may occur in diverse organs over time [1]. Though these tumors are highly variable in location (e.g., colorectal, genitourinary, endometrium, pancreas, hepatobiliary tract, brain, cervix, breast, blood, and lung [1]), duodenal cancers are rarely reported. The two special features of our case are thus centered around diagnosis and preservation-focused treatment. First, although the tumor was located in the 3rd portion of the duodenum, which presents screening difficulties, previous MTS diagnosis could have simplified detection. Second, despite advanced status, reduced radical surgery was performed and the patient has since been free from both recurrence and metastasis.

The diagnosis of MTS usually takes the form of one of the following: a cutaneous sebaceous adenoma, sebaceous epithelioma, sebaceous carcinoma, or a keratoacanthoma with sebaceous differentiation, plus at least one visceral malignancy, in the absence of another probable causes (e.g., radiotherapy or acquired immune deficiency syndrome) (Table 1) [2]. Although her past history fulfilled the MTS diagnostic criteria, diagnosis had not occurred for our patient over decades. However, Mayo risk scoring, with a score of 3 (specificity of 98%), for Lynch syndrome would have been applicable in our case to predict germline mutations in mismatch repair genes when diagnosing the sebaceous tumor (Table 2) [6]. Thus, as MTS is a subtype of Lynch syndrome, earlier diagnosis might have been possible if it was suspected, but unawareness of MTS may have precipitated the extended delay in diagnosis.

**Table 1:** Diagnostic criteria for Muir–Torre syndrome

Group A	Sebaceous adenoma
	Sebaceous epithelioma
	Sebaceous carcinoma
	Keratoacanthoma with sebaceous differentiation
Group B	Visceral malignancy
Group C	Multiple keratoacanthomas
	Multiple visceral malignancies
	Family history of Muir–Torre syndrome

Diagnosis requires one criterion from group A and group B, or all three from group C, in absence of other predisposing factors, such as radiotherapy for childhood retinoblastoma associated with eyelid sebaceous carcinoma, AIDS, and Kaposi’s sarcoma (in which case neither the Kaposi’s sarcoma nor a lymphoma should count as the visceral malignancy), or nevus sebaceous (in which neoplasms such as sebaceous epithelioma are predisposed to develop)

**Table 2:** Mayo Muir–Torre syndrome risk score

Variable	Score
Age at sebaceous neoplasm diagnosis (years)	
60 or older	0
Younger than 60	1
Total number of sebaceous neoplasms	
1	0
2 or more	2
Personal history of any Lynch-related cancer	
No	0
Yes	1
Family history of any Lynch-related cancer	
No	0
Yes	1

Scores for the four variables are summed to create a total score, the “Mayo MTS risk score”, with a possible range of 0–5. A score of 3 or more has a sensitivity of 70% and specificity of 98% for predicting a germline mutation in a Lynch syndrome mismatch repair gene

Although the carcinoma was advanced, its location in the duodenal third portion, as well as preservation of the papilla of Vater, led us to choose partial duodenectomy over pancreatoduodenectomy. In general, for patients with T2–4 duodenal carcinoma, pancreatoduodenectomy with regional lymph node dissection is recommended [7]. On the other hand, for T1a duodenal carcinoma and benign tumors (e.g., duodenal polyposis), partial duodenectomy and pancreas-sparing duodenectomy (PSD) are considered sufficient if the papilla of Vater is preserved [7, 8]. Our patient had no recurrence or metastases although her duodenal adenocarcinoma was pathologically T3N0M0. As for visceral malignancies in MTS patients, prognoses are usually favorable since they typically have low invasiveness and aggressiveness [2]. As such, there is the possibility to avoid pancreatomy and rely on partial duodenectomy plus PSD for duodenal advanced carcinoma in MTS patients. Since these patients will require extensive treatment, a function-sparing strategy improves quality of life and supports long-term follow-up.

We used keywords “muir-torre syndrome” and “duodenal carcinoma” in Pubmed searches, finding 5 reports in addition to our case (Table 3) [4, 9–12]. Demographically, the reported ratio of men to women is 4:2 with a mean age of 58.6 years old. Sebaceous neoplasms were localized to the shoulder in 2 of 6 patients, face only in 3 of 6 patients, and face, back, and head in 1 of 6 patients. Two of the 6 reported patients had a visceral malignancy only in the duodenum while 4 other patients had simultaneous colon, rectal, gastric, bladder, prostate or larynx malignancies. One of the 6 patients experienced duodenal carcinoma before detection of a sebaceous neoplasm, but 5 other patients had the neoplasm occur after carcinoma detection. Akhtar et al. reported that internal malignancies appeared before sebaceous tumors in 56% of cases, concurrently in 6% of cases, and after in 22% of cases while 16% of cases demonstrated no temporal relationship in MTS patients [11]. Duodenal carcinoma in MTS patients may thus manifest after the appearance of sebaceous tumors.

**Table 3:** Cases of Muir–Torre syndrome accompanied with duodenal carcinoma

Authors	Age/sex	Sebaceous tumor	Location of duodenal carcinoma	Surgery	Abdominal symptoms	Appearance order of duodenal carcinoma to sebaceous tumor	Genetic disorder
Our case	58/F	Shoulder	3^rd^ portion	Partial duodenectomy	Pain, nausea, anorexia	After	MSH2
Matthews [12]	64/M	Shoulder	Ampulla of Vater	PD	Jaundice	After	UNK
Akhtar [11]	66/M	Face, back, head	3rd portion	Partial duodenectomy	None	After	MLH1 MLH2
Narita [10]	69/M	Face	UNK	UNK	UNK	After	UNK
Donati [9]	53/F	Face	UNK	UNK	Pain, vomiting	Before	UNK
Muir [4]	42/M	Face	UNK	PD	Pain, jaundice	After	UNK

*M* male, *F* female, *UNK* unknown, *PD* pancreatoduodenectomy

Genetic disorders were elucidated in 2 of 6 cases, including our case. The genetic features of MTS accompanying duodenal carcinoma are therefore difficult to extensively discuss due to the scarcity of reported cases. Once a diagnosis of MTS is made, regular and complete examinations for gastrointestinal and genitourinary cancers are crucial because MTS is an autosomal-dominant disease with risk of multiple primary malignancies over time [2, 13]. For this purpose, Ishiguro et al. reported that positron emission tomography/computed tomography was useful for the diagnosis and follow-up of MTS-related internal malignancies [14]. If our case had been diagnosed with MTS at the time of the shoulder sebaceous carcinoma, the duodenal carcinoma might have been detected much earlier. This reinforces the importance of being proactive in regular cancer screening tests and highlighting abdominal symptoms as markers for initiating multimodal gastrointestinal cancer searches. Finally, as concomitant skin sebaceous tumors and visceral malignancies are suggestive of MTS, genetic screening test are strongly recommended in these cases.

## Conclusions

Here we reported an advanced duodenal carcinoma initially diagnosed as supra-mesenteric artery syndrome in a patient with MTS, who was freed from both recurrence and metastasis after reduced radical surgery. Any patient presenting with both a skin sebaceous tumor and visceral malignancy should be screened for MTS. Finally, the propensity for multiple gastrointestinal cancers in some MTS patients warrants function-preserving surgical strategies.

## Data Availability

No additional data.

## Abbreviations

MTS: Muir–Torre syndrome
BMI: Body mass index
MMR: Mismatch repair
PSD: Pancreas-sparing duodenectomy
